# Supplementary material for: Spatiotemporal Detection of Unusual Human Population Behavior Using Mobile Phone Data
Source: PLoS One. 2015 Mar 25;10(3):e0120449. doi: 10.1371/journal.pone.0120449 (PMC4373934; doi:10.1371/journal.pone.0120449)
Supplement: S1 Supporting Information — (PDF) [file pone.0120449.s001.pdf]

SUPPORTING INFORMATION FOR "SPATIOTEMPORAL DETECTION OF UNUSUAL HUMAN POPULATION BEHAVIOR USING MOBILE PHONE DATA"

ADRIAN DOBRA, NATHALIE E. WILLIAMS AND NATHAN EAGLE

Contents

List of Figures 1
SI1: The road network and the grid cell system 5
SI1.1: The Road Network System 5
SI1.2: The Grid Cell System 5
SI2: Measures of human behavior 7
SI3: Identifying days with anomalous human behavior at one site 8
References 9

List of Figures

A **Rwandan road network system**. Map of Rwanda showing the position of the 269 cellular towers (red) and the structure of the network of roads that are also segments in quickest routes (blue). 6

B **Rwandan grid cell system**. Map of Rwanda showing the position of the cellular towers (red) with respect to the 2040 5 km x 5 km grid cells. Rwanda's boundary is shown in blue. 7

C **Daily time series of the size of the largest spatial clusters of sites with high call volume and movement frequency.** For each day, we show the maximum number of sites that belong to the same spatial cluster and recorded higher than usual call volume and movement frequency. Red indicates days for which the largest spatial cluster has at least two sites, while blue indicates days for which the largest spatial cluster has one site. 10

D **Daily time series of the size of the largest spatial clusters of sites with low call volume and movement frequency.** For each day, we show the maximum number of sites that belong to the same spatial cluster and recorded lower than usual call volume and movement frequency. Red indicates days for which the largest spatial cluster has at least two sites, while blue indicates days for which the largest spatial cluster has one site. 10

E **Daily time series of the size of the largest spatial clusters of sites with high call volumes.** For each day, we show the maximum number of sites that belong to the same spatial cluster and recorded higher than usual call volume. Red indicates days for which the largest spatial cluster has at least two sites, while blue indicates days for which the largest spatial cluster has one site. 11

F **Daily time series of the size of the largest spatial clusters of sites with low call volume.** For each day, we show the maximum number of sites that belong to the same spatial cluster and recorded lower than usual call volume. Red indicates days for which the largest spatial cluster has at least two sites, while blue indicates days for which the largest spatial cluster has one site. 11

G **Daily time series of the size of the largest spatial clusters of sites with high movement frequencies.** For each day, we show the maximum number of sites that belong to the same spatial cluster and recorded higher than usual movement frequency. Red indicates days for which the largest spatial cluster has at least two sites, while blue indicates days for which the largest spatial cluster has one site. 12

H **Daily time series of the size of the largest spatial clusters of sites with low movement frequency.** For each day, we show the maximum number of sites that belong to the same spatial cluster and recorded lower than usual movement frequency. Red indicates days for which the largest spatial cluster has at least two sites, while blue indicates days for which the largest spatial cluster has one site. 12

I **Sites with unusually high behavior on September 17, 2005.** Four sites recorded unusually high call volume and movement frequency, and belong to the same spatial cluster. This anomalous pattern of communication was potentially caused by an ACLED event recorded on September 16, 2005. The green cross marks the reported location of this event, while the two green circles mark the 25 and 50 km areas around this location. The distance between the event's location and the centroid of the closest site with unusual communication activity is 1.7 km. 13

J **Sites with unusually low behavior on January 15, 2006.** Nine sites recorded unusually low call volume and movement frequency. Seven additional sites recorded unusually low call volume. Most of the sites in these two groups belong to one spatial cluster. One spatial cluster with only one site is also present. This anomalous pattern of communication was potentially caused by an ACLED event recorded on the same day. The green cross marks the reported location of this event, while the two green circles mark the 25 and 50 km areas around this location. The distance between the event's location and the centroid of the closest site with unusual call volume (movement frequency) is 3.8 (5.4) km. 14

K **Sites with unusually low behavior on November 26, 2006.** Seven sites recorded unusually low call volume and movement frequency. Seven additional sites recorded unusually low movement frequency. Most of the sites in these two groups belong to one spatial cluster. One spatial cluster with only one site is

also present. This anomalous pattern of communication was potentially caused by an ACLED event recorded on November 25, 2006. The green cross marks the reported location of the event, while the two green circles mark the 25 and 50 km areas around this location. The distance between the event's location and the centroid of the closest site with unusual call volume (movement frequency) is 3.38 (1.83) km. 15

L **Sites with unusually low behavior on November 19, 2008.** A number of 45 sites recorded unusually low call volume and movement frequency. Four additional sites recorded unusually low call volume. The sites in these two groups belong to one spatial cluster. This anomalous pattern of communication was potentially caused by an ACLED event recorded on the same day. The green cross marks the reported location of this event, while the two green circles mark the 25 and 50 km areas around this location. The distance between the event's location and the centroid of the closest site with unusual call volume and movement frequency is 1.8 km. 16

M **Sites with unusually low behavior on September 19, 2007.** A number of 53 sites recorded unusually low call volume and movement frequency. These sites belong to the same spatial cluster. 17

N **Sites with unusually high behavior on December 24, 2007.** A number of 26 sites recorded unusually high call volume and movement frequency. A number of 21 additional sites recorded unusually high call volume, while one other site recorded unusually high movement frequency. The sites in these three groups belong to one or two spatial clusters. 18

O **Sites with unusually high behavior on December 24, 2008.** A number of 59 sites recorded unusually high call volume and movement frequency. A number of 17 additional sites recorded unusually high call volume, while two other sites recorded unusually high movement frequency. Most of the sites in these three groups belong to one or two spatial clusters, but small spatial clusters with only one site are also present. 19

P **Sites with unusually high behavior on January 1, 2008.** A number of 21 sites recorded unusually high call volume and movement frequency. One additional site recorded unusually high call volume, while 30 other sites recorded unusually high movement frequency. Most of the sites in these three groups belong to one spatial cluster. Smaller spatial clusters with up to four sites are also present. 20

Q **Sites with unusually high behavior on December 31, 2008.** A number of 28 sites recorded unusually high call volume and movement frequency. A number of 22 additional sites recorded unusually high call volume. Most of the sites in these three groups belong to one or two spatial clusters. Smaller spatial clusters with one or two sites are also present. 21

R **Sites with unusually high behavior on January 1, 2009.** A number of 20 sites recorded unusually high call volume and movement frequency. One additional

site recorded unusually high call volume, while 35 other sites recorded unusually high movement frequency. Most of the sites in these three groups belong to one spatial cluster. Smaller spatial clusters with one or two sites are also present. 22

S **Sites with unusually high behavior on November 9, 2007.** A number of 52 sites recorded unusually high call volume and movement frequency. Three additional sites recorded unusually high call volume, while one other site recorded unusually high movement frequency. The sites in these three groups belong to one spatial cluster. 23

T **Sites with unusually low behavior on April 24, 2008.** A number of 61 sites recorded unusually low call volume and movement frequency. Five additional sites recorded unusually low call volume, while one other site recorded unusually low movement frequency. The sites in these three groups belong to one spatial cluster. 24

U **Sites with unusually low behavior on April 25, 2008.** A number of 53 sites recorded unusually low call volume and movement frequency. Two additional sites recorded unusually low movement frequency. The sites in these two groups belong to one large spatial cluster and to another small spatial cluster with just one site. 25

V **Sites with unusually low behavior on April 7, 2007.** A number of 26 sites recorded unusually low call volume and movement frequency. Five additional sites recorded unusually low call volume, while 7 other sites recorded unusually low movement frequency. Most of the sites in these three groups belong to two spatial clusters. Smaller spatial clusters are also present. 26

W **Sites with unusually low behavior on April 8, 2007.** Four sites recorded unusually low call volume and movement frequency. Three additional sites recorded unusually low call volume, while one other site recorded unusually low movement frequency. The sites in these three groups belong to spatial clusters of size 1 or 2. 27

X **Sites with unusually low behavior on April 7, 2008.** A number of 24 sites recorded unusually low call volume and movement frequency. Twelve additional sites recorded unusually low call volume, while 3 other sites recorded unusually low movement frequency. Most of the sites in these three groups belong to one spatial cluster. Smaller spatial clusters are also present. 28

Y **Sites with unusually low behavior on April 8, 2008.** Four sites recorded unusually low call volume and movement frequency. A number of 15 additional sites recorded unusually low movement frequency. Most of the sites in these two groups belong to one spatial cluster. Smaller spatial clusters are also present. 29

SI1: The road network and the grid cell system

The methodology presented in this paper is based on two Geographic Information Systems (GIS) components: a road network system for Rwanda and a grid cell system which divides a spatial bounding box for Rwanda's boundary into 2040 5 km x 5 km cells. Figs. A and B display the locations of the 269 cellular towers that appear in the Rwandan CDR data with respect to the road network and the grid cell system, respectively. The grid cells that contain at least one tower are called sites. Only 155 out of the 2040 grid cells are sites. Four sites in the Kigali area contain the largest number of cellular towers: 41, 22, 6 and 5, respectively. Seven sites contain four towers, four sites contain three towers, 14 sites contain two towers and the other sites contain only one tower. These counts represent the towers that belong to a site between June 1, 2005 and January 1, 2009. In any period of time between these dates, all, some or none of the towers that belong to a site are actually active (i.e. handle cellular communications). As such, the number of sites (i.e., grid cells that contain at least one active tower) at any time might be smaller than 155.

SI1.1: The Road Network System. We use road network data from the crowd sourced OpenStreetMap[1]. Roads are categorized with respect to their quality in the following hierarchy: trunk roads, primary roads, secondary roads and tertiary roads. We estimate that the average speeds of travel for these four types of roads are 120 km/h, 60 km/h, 45 km/h and 30 km/h, respectively. Based on this determination, we employed ESRI's ArcGIS[2] to determine approximate travel distances and travel times between the centroids of pairs of sites. We used the function "Closest Facility" of ArcGIS Network Analyst[3] to identify the quickest road paths between the centroids of any pair of sites and stored these $\binom{155}{2} = 23870$ routes together with their corresponding travel distances and travel times. We also identify the sites on the quickest route between the centroids of each pair of sites.

SI1.2: The Grid Cell System. We overlay a customized rectangular grid with square cells of equal size on the map of Rwanda, and replace cellular tower locations with the centroid of the sites they belong to. Instead of measuring straight line distances from tower to tower, we measure distances between the centroids of the sites via the quickest road route which connects these centroids. The raw road network data downloaded from OSM was such that 11 sites were not intersected by the Rwandan road network. To connect these sites to the road network, we moved the location of their centroids to adjacent grid cell centroids.

Choosing the size of the grid cells is an important decision. Based on geographical and technological considerations, we estimated catchment areas in which a user of a cellular tower is likely to be located. We estimate that the maximum signal distance for the type of towers in Rwanda is roughly 10 km. Several factors further reduce this maximum signal distance, including relative location of a user with respect to a tower, topography of the areas surrounding towers, and the decay in signal strength with increasing distances from

[1] `http://www.openstreetmap.org/`

[2] `http://www.esri.com/software/arcgis`

[3] `http://www.esri.com/software/arcgis/extensions/networkanalyst`

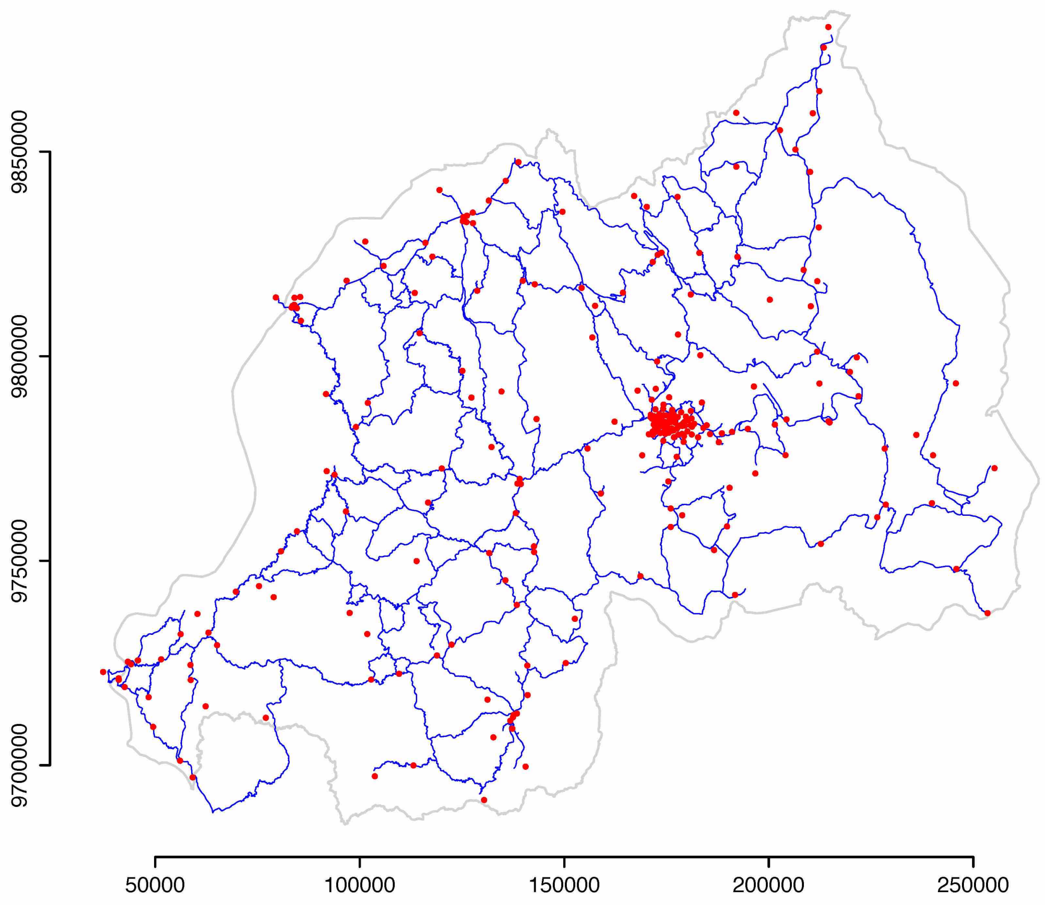

Fig. A. **Rwandan road network system**. Map of Rwanda showing the position of the 269 cellular towers (red) and the structure of the network of roads that are also segments in quickest routes (blue).

towers. As such, we reduce the maximum user-to-tower distance to 5 km. The resulting 5 km x 5 km grid cell system is a 51 x 40 matrix (2040 grid cells) that covers 51,000 km^2 extending just outside of the border of Rwanda – see Fig. B. Each grid cell is indexed by a number from 1 to 2040: grid cell 1 is located in the lower left corner and grid cell 2040 is located in the upper right corner. The indices increase first by row, then by column. Each of the 269 cellular towers is subsequently mapped to its corresponding grid cell (site).

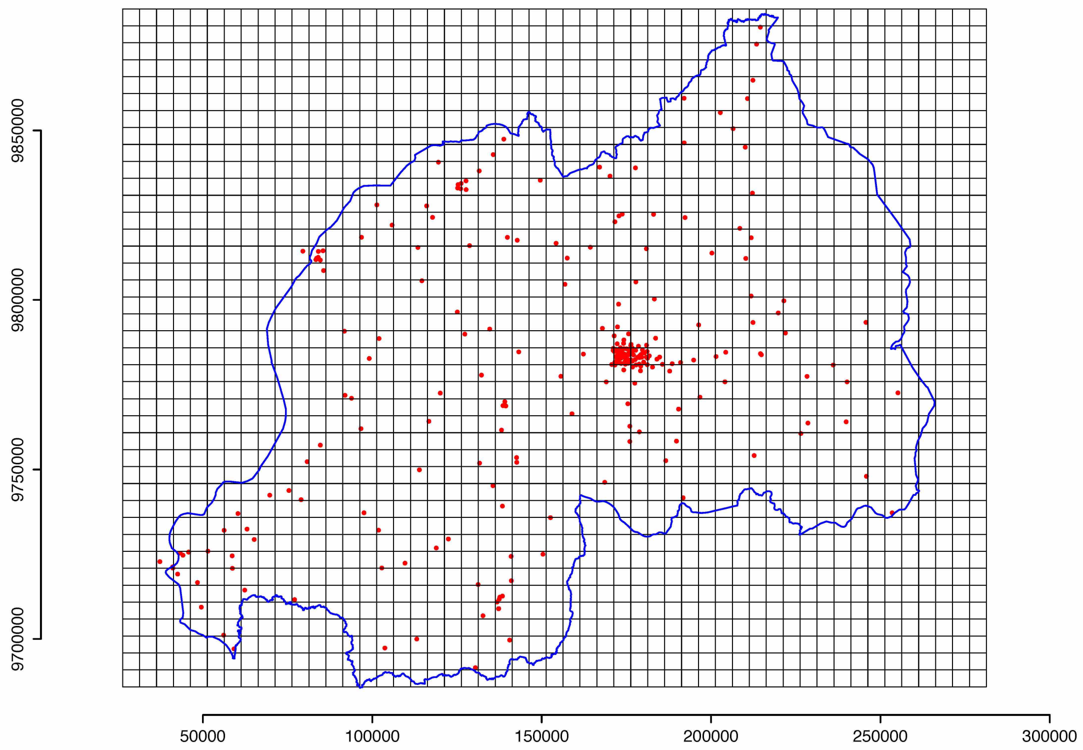

Fig. B. **Rwandan grid cell system**. Map of Rwanda showing the position of the cellular towers (red) with respect to the 2040 5 km x 5 km grid cells. Rwanda's boundary is shown in blue.

SI2: Measures of human behavior

Consider the sequence of CDRs associated with a mobile phone in a reference period of time $\mathcal{T}$ (e.g., a day, a week, a month or a year):

$$M = \{m_1, m_2, \ldots, m_n\}. \quad \text{(S1)}$$

We assume that the wireless-service provider that generated these CDRs has K active towers in the reference time period $\mathcal{T}$, and that the spatial locations l_i^{CT}, $i \in \mathcal{K} = \{1, 2, \ldots, K\}$ of these active towers are known. In Equation (S1), $m_i \in \mathcal{K}$, $1 \leq i \leq n$, is the identifier of the cellular tower that handled the communication represented by the i-th CDR in the sequence. If $i < j$ the communication represented by m_i was recorded before the communication represented by m_j. We refer to M as the spatiotemporal trajectory of the cellular phone that generated the sequence of CDRs. We assume that the region of interest

was divided into non-overlapping grid cells identified by indices in $\mathcal{Q} \in \{1, 2, \ldots, Q\}$. We denote by l_j^{GC} the location of the centroid of the grid cell $j \in \mathcal{Q}$. We introduce a mapping function $q^{GC}(\cdot)$ which gives, for each cellular tower $i \in \mathcal{K}$, the grid cell $q^{GC}(i) \in \mathcal{Q}$ the tower belongs to. The sites are those grid cells that contain at least one tower:

$$\mathcal{S} = \left\{ j : j \in \mathcal{Q} \text{ such that there exists } i \in \mathcal{K} \text{ with } q^{GC}(i) = j \right\}.$$

Since we assume that all the towers indexed by $\mathcal{K}$ are active in the reference time period $\mathcal{T}$, $\mathcal{S}$ represents the set of sites in $\mathcal{T}$.

We transform the spatiotemporal trajectory M from Equation (S1) into the time ordered sequence of sites to which the active towers that appear in M belong to:

$$M^{GC} = \{g_1, g_2, \ldots, g_n\},$$

where $g_i = q^{GC}(m_i) \in \mathcal{S}$.

The measure of behavior called "call volume" is the number of times a person communicates in the reference time period $\mathcal{T}$. For the spatiotemporal trajectory M^{GC}, this measure is equal with the length of the sequence n.

The measure of behavior called "movement frequency" (also referred to as "number of trips" in [1]) is a count of the number of times a person communicates from a different grid cell than their previous communication:

$$\# \{ i : i \in \{1, 2, \ldots, n-1\} \text{ such that } g_i \neq g_{i+1} \}.$$

To see why the movement frequency captures an aspect of human behavior complementary to the call volume, consider an example person who makes 10 calls from one site and another example person that calls once from 10 different sites. The call volumes of the two persons are equal. But the movement frequency of the first person is 0, while the movement frequency of the second person is 9. The behaviors of these two persons are dissimilar: the first one makes multiple calls from one site and is not mobile, while the second person moves significantly more. In this paper we employ only one measure of mobility, but combinations of several measures of mobility can also be explored. See [1] for an in-depth discussion of measures of mobility constructed from mobile phone records.

SI3: Identifying days with anomalous human behavior at one site

We consider a reference time period P of T consecutive days. We denote by $Y_{i,t}$ the behavioral measurement associated with the i-th person that made at least one call from a site S during day t with $i \in \{1, 2, \ldots, n_t\}$. Since $Y_{i,t}$ represent either counts of the number of calls or counts of the number of trips, we assume Poisson sampling models for measurements within each day:

$$Y_{1,t}, Y_{2,t}, \ldots, Y_{n_t,t} \mid \theta_t \sim \text{ i.i.d. } \mathsf{Poi}(\theta_t).$$

The Poisson means $\{\theta_t : t \in \mathrm{P}\}$ have independent Gamma prior distributions $\mathsf{G}(a, b)$. The shape parameter a and the rate parameter b are set to 1, and yield proper priors with mode equal to 0, and with mean and variance equal with 1. Therefore, a priori, we assume that

individuals make 1 call and make 1 trip in any given day. The posterior distribution of θ_t is also Gamma:

$$\theta_t \mid Y_{1,t}, Y_{2,t}, \dots, Y_{n_t,t} \sim \mathsf{G}\left(a + \sum_{i=1}^{n_t} Y_{i,t}, b + n_t\right).$$

The rate parameter b is interpreted as the number of prior observations. Given that every day hundreds or thousands of people make calls from each site, the behavioral measurements associated with each day and each site have a large weight in the posterior distributions of the Poisson means θ_t with respect to the Gamma priors. Predictions about the measurement $\tilde{Y}_t$ of a new person that calls from site S during day t which account for uncertainty about the Poisson means are obtained based on the predictive distribution of $\tilde{Y}_t$ that is a negative binomial

$$\tilde{Y}_t \mid Y_{1,t}, Y_{2,t}, \dots, Y_{n_t,t} \sim \mathsf{NegBin}\left(a + \sum_{i=1}^{n_t} Y_{i,t}, b + n_t\right). \tag{S2}$$

A Monte Carlo estimate of the probability that a random caller from day t_0 in the time period P had a larger behavioral measurement (i.e., made more calls or moved more frequently) than a random caller from a random day in P other than t_0 is obtained by repeating the following steps $N = 10000$ times. We work with a counter l_{t_0} initialized at 0.

(1) For each day t in the reference time period P simulate $\tilde{Y}_t$ from the negative binomial predictive distribution given in Equation (S2).
(2) Sample a day t_1 in $\mathrm{P} \setminus \{t_0\}$.
(3) If $\tilde{Y}_{t_0} > \tilde{Y}_{t_1}$ increment l_{t_0} by 1.

The Monte Carlo estimate is given by l_{t_0}/N.

References

[1] Williams NE, Thomas TA, Dunbar M, Eagle N, Dobra A (2014) Measures of human mobility using mobile phone records enhanced with GIS data. Available on http://arxiv.org/abs/1408.5420.

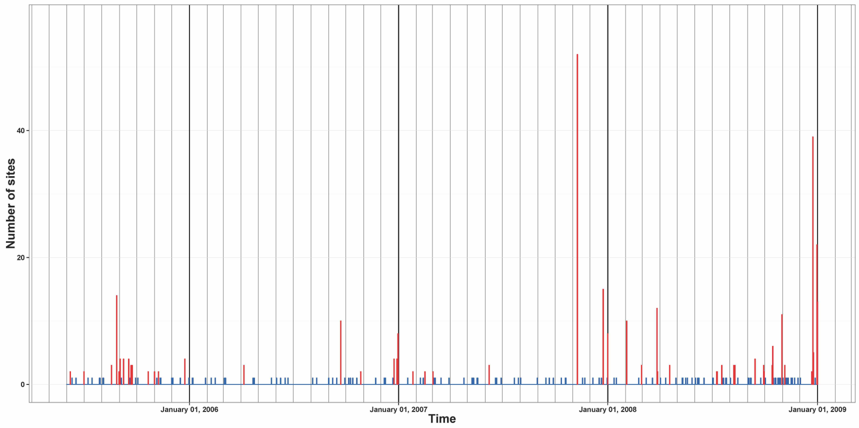

Fig. C. **Daily time series of the size of the largest spatial clusters of sites with high call volume and movement frequency.** For each day, we show the maximum number of sites that belong to the same spatial cluster and recorded higher than usual call volume and movement frequency. Red indicates days for which the largest spatial cluster has at least two sites, while blue indicates days for which the largest spatial cluster has one site.

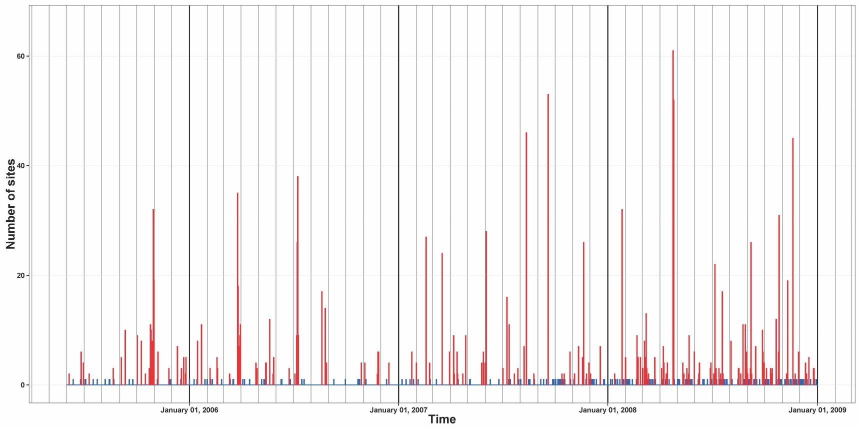

Fig. D. **Daily time series of the size of the largest spatial clusters of sites with low call volume and movement frequency.** For each day, we show the maximum number of sites that belong to the same spatial cluster and recorded lower than usual call volume and movement frequency. Red indicates days for which the largest spatial cluster has at least two sites, while blue indicates days for which the largest spatial cluster has one site.

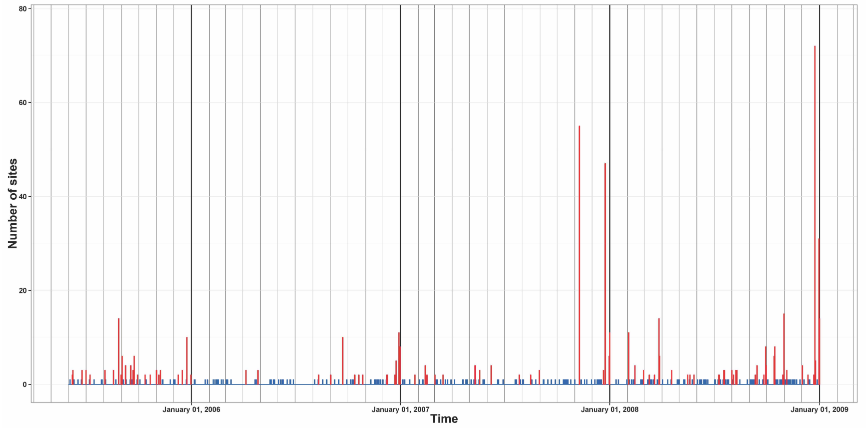

Fig. E. **Daily time series of the size of the largest spatial clusters of sites with high call volumes.** For each day, we show the maximum number of sites that belong to the same spatial cluster and recorded higher than usual call volume. Red indicates days for which the largest spatial cluster has at least two sites, while blue indicates days for which the largest spatial cluster has one site.

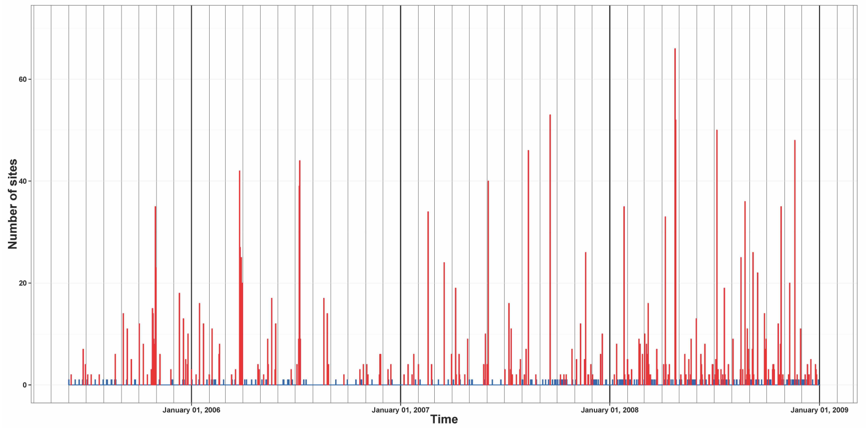

Fig. F. **Daily time series of the size of the largest spatial clusters of sites with low call volume.** For each day, we show the maximum number of sites that belong to the same spatial cluster and recorded lower than usual call volume. Red indicates days for which the largest spatial cluster has at least two sites, while blue indicates days for which the largest spatial cluster has one site.

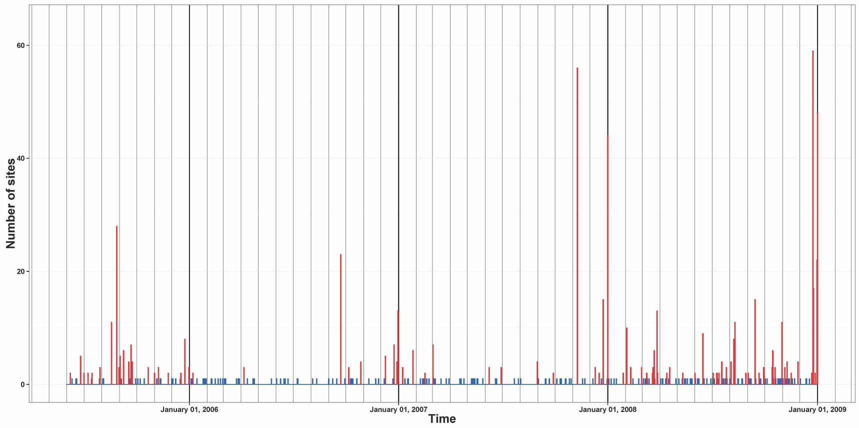

Fig. G. **Daily time series of the size of the largest spatial clusters of sites with high movement frequencies.** For each day, we show the maximum number of sites that belong to the same spatial cluster and recorded higher than usual movement frequency. Red indicates days for which the largest spatial cluster has at least two sites, while blue indicates days for which the largest spatial cluster has one site.

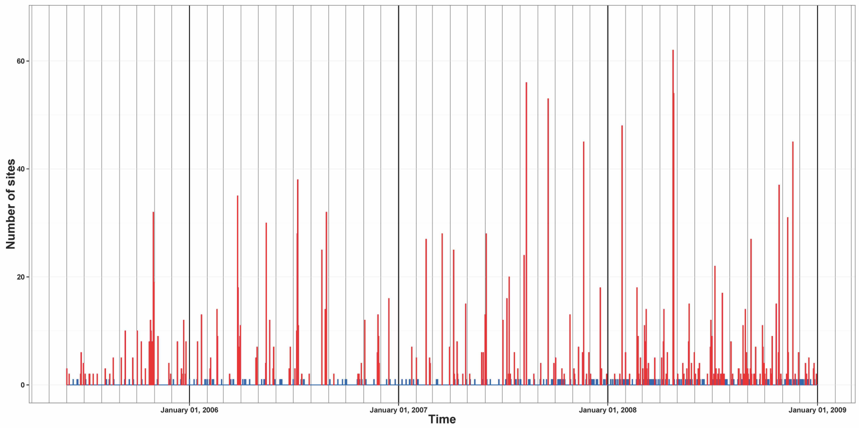

Fig. H. **Daily time series of the size of the largest spatial clusters of sites with low movement frequency.** For each day, we show the maximum number of sites that belong to the same spatial cluster and recorded lower than usual movement frequency. Red indicates days for which the largest spatial cluster has at least two sites, while blue indicates days for which the largest spatial cluster has one site.

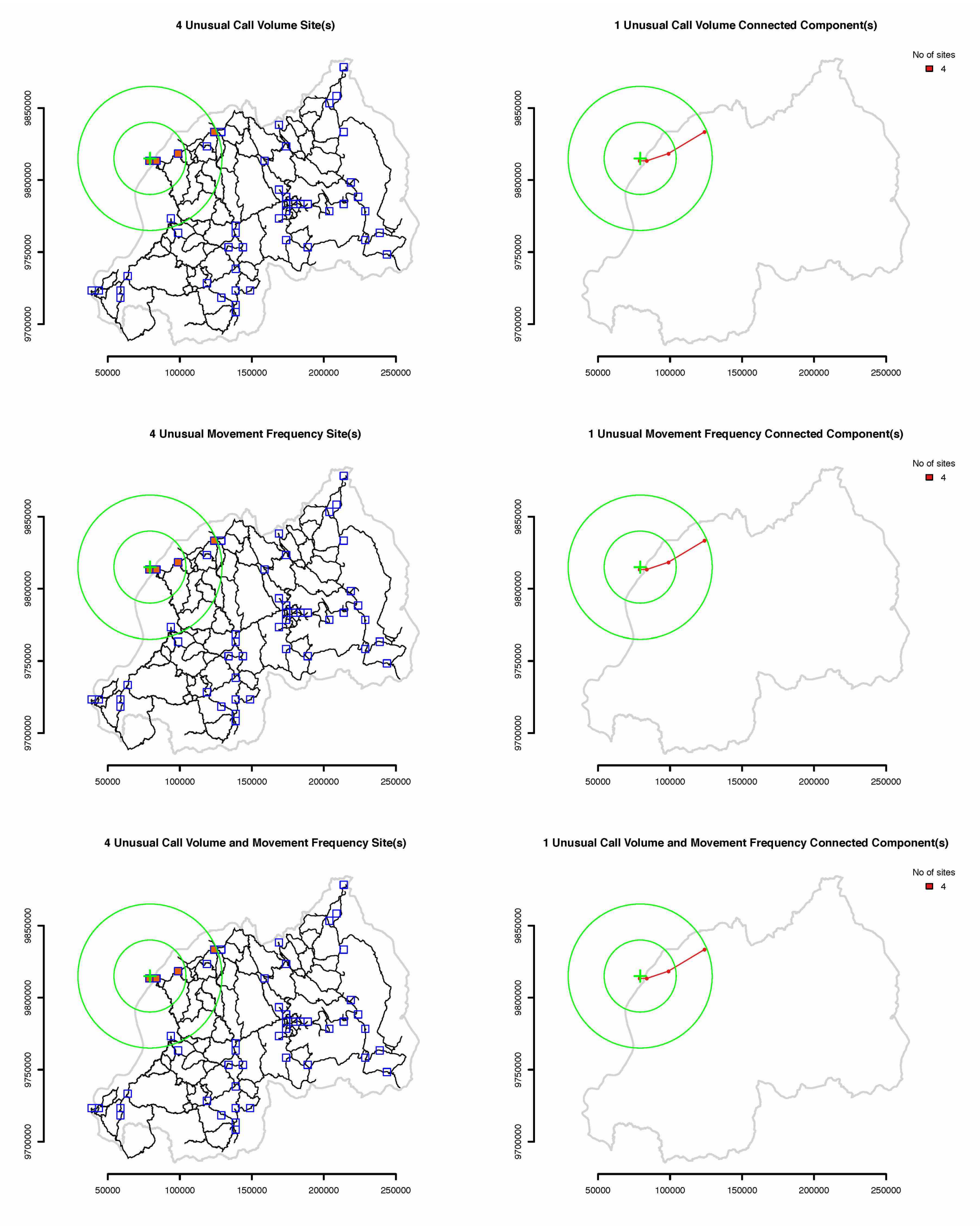

FIG. I. **Sites with unusually high behavior on September 17, 2005.** Four sites recorded unusually high call volume and movement frequency, and belong to the same spatial cluster. This anomalous pattern of communication was potentially caused by an ACLED event recorded on September 16, 2005. The green cross marks the reported location of this event, while the two green circles mark the 25 and 50 km areas around this location. The distance between the event's location and the centroid of the closest site with unusual communication activity is 1.7 km.

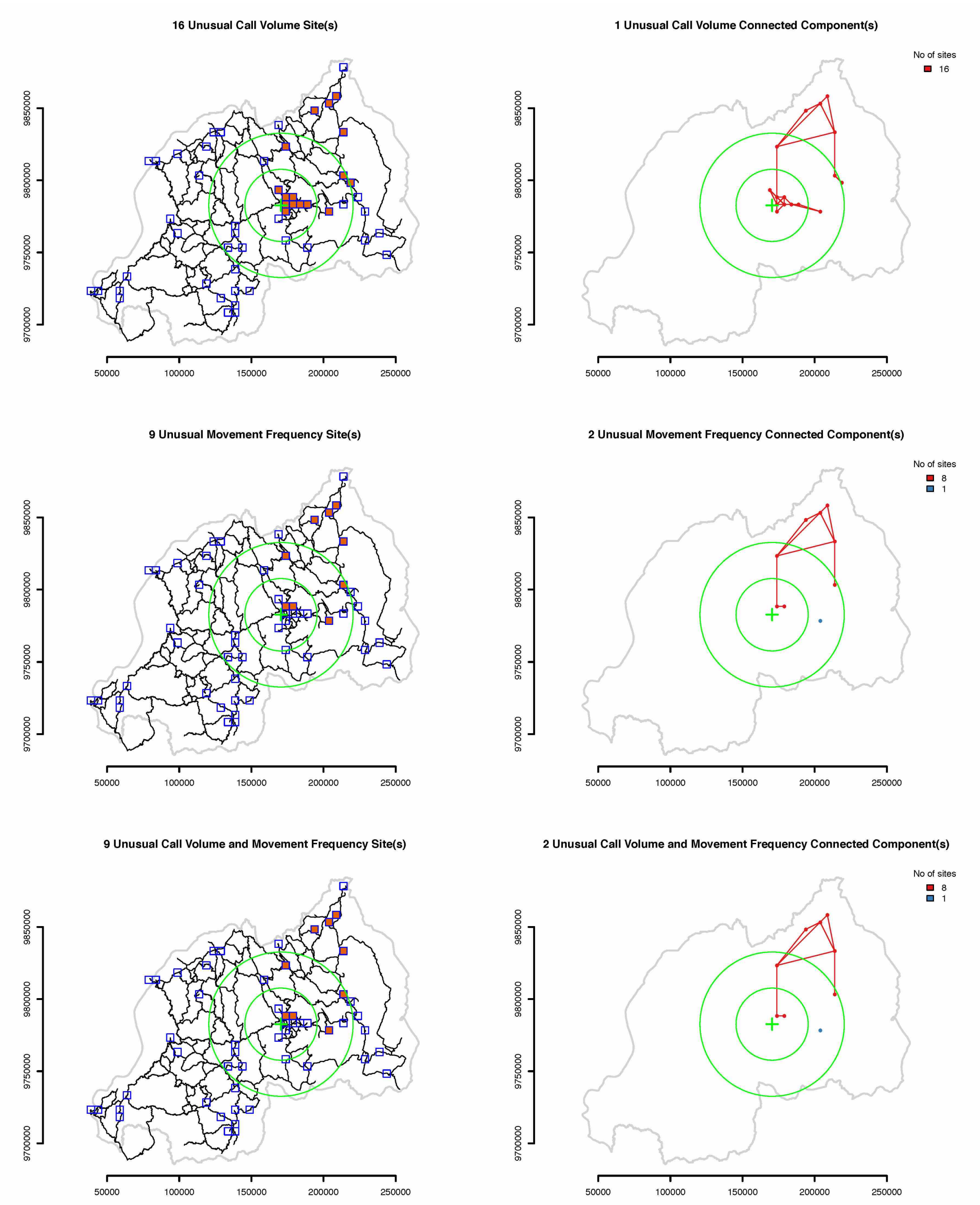

Fig. J. **Sites with unusually low behavior on January 15, 2006.** Nine sites recorded unusually low call volume and movement frequency. Seven additional sites recorded unusually low call volume. Most of the sites in these two groups belong to one spatial cluster. One spatial cluster with only one site is also present. This anomalous pattern of communication was potentially caused by an ACLED event recorded on the same day. The green cross marks the reported location of this event, while the two green circles mark the 25 and 50 km areas around this location. The distance between the event's location and the centroid of the closest site with unusual call volume (movement frequency) is 3.8 (5.4) km.

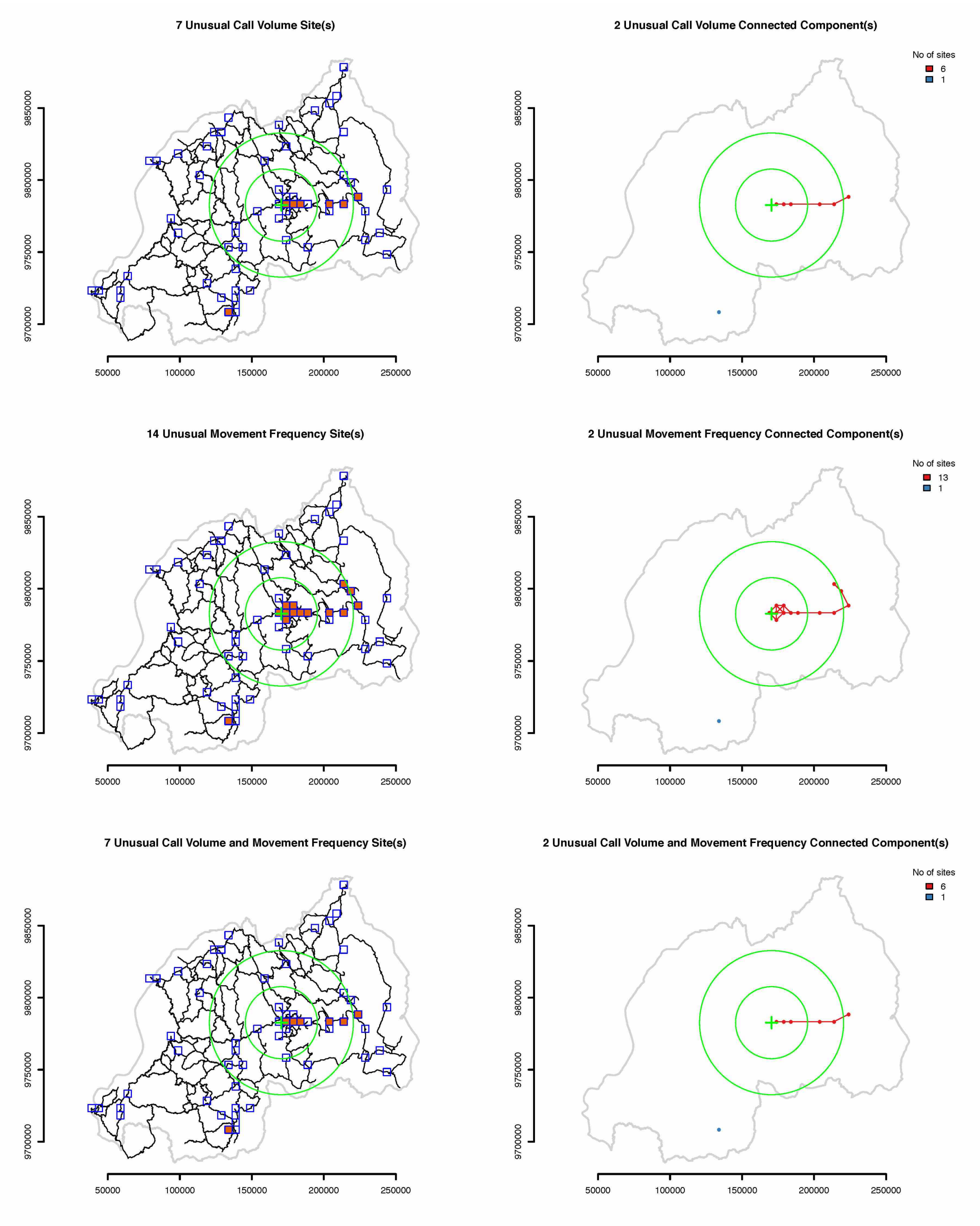

Fig. K. **Sites with unusually low behavior on November 26, 2006.** Seven sites recorded unusually low call volume and movement frequency. Seven additional sites recorded unusually low movement frequency. Most of the sites in these two groups belong to one spatial cluster. One spatial cluster with only one site is also present. This anomalous pattern of communication was potentially caused by an ACLED event recorded on November 25, 2006. The green cross marks the reported location of the event, while the two green circles mark the 25 and 50 km areas around this location. The distance between the event's location and the centroid of the closest site with unusual call volume (movement frequency) is 3.38 (1.83) km.

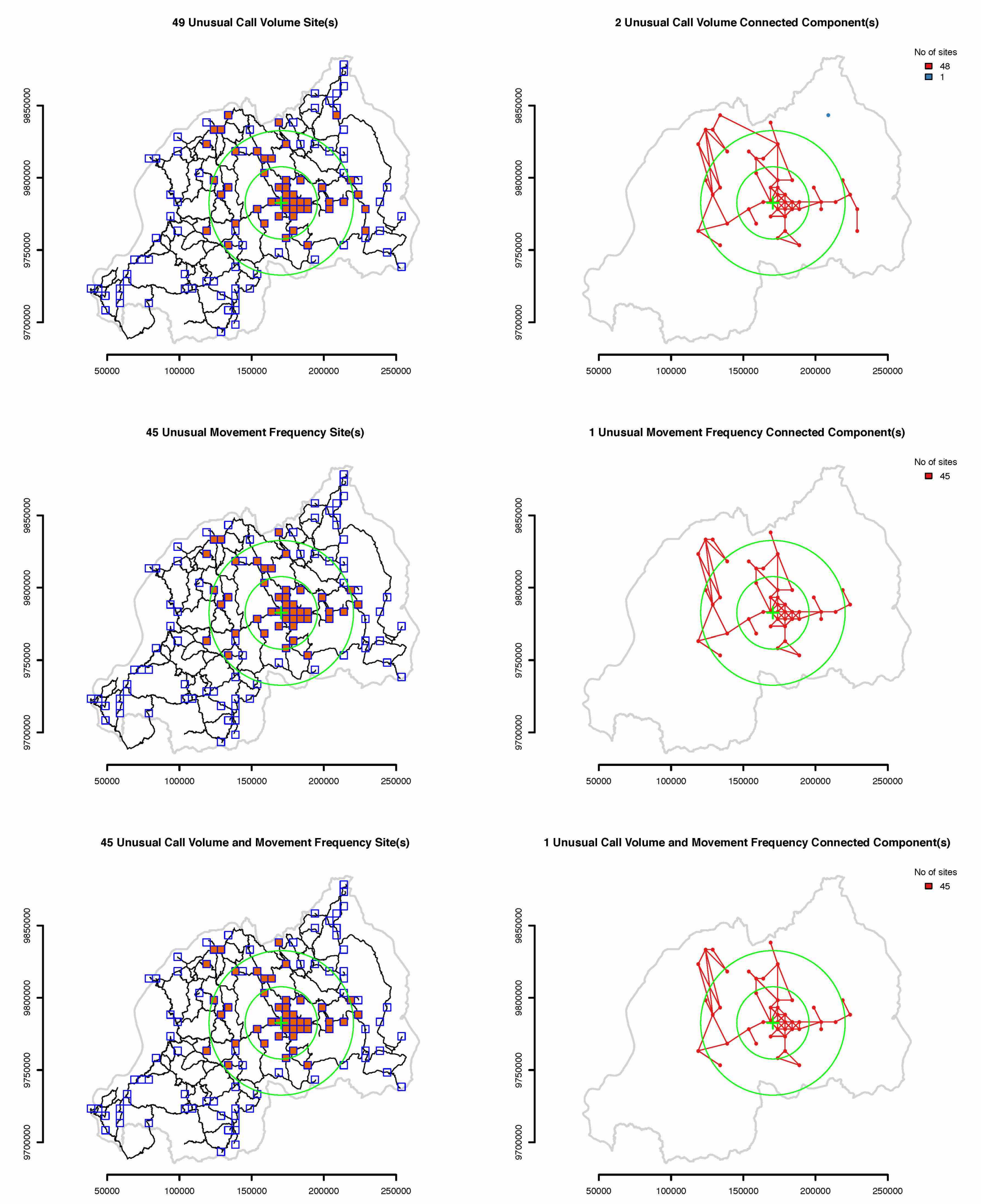

Fig. L. **Sites with unusually low behavior on November 19, 2008.** A number of 45 sites recorded unusually low call volume and movement frequency. Four additional sites recorded unusually low call volume. The sites in these two groups belong to one spatial cluster. This anomalous pattern of communication was potentially caused by an ACLED event recorded on the same day. The green cross marks the reported location of this event, while the two green circles mark the 25 and 50 km areas around this location. The distance between the event's location and the centroid of the closest site with unusual call volume and movement frequency is 1.8 km.

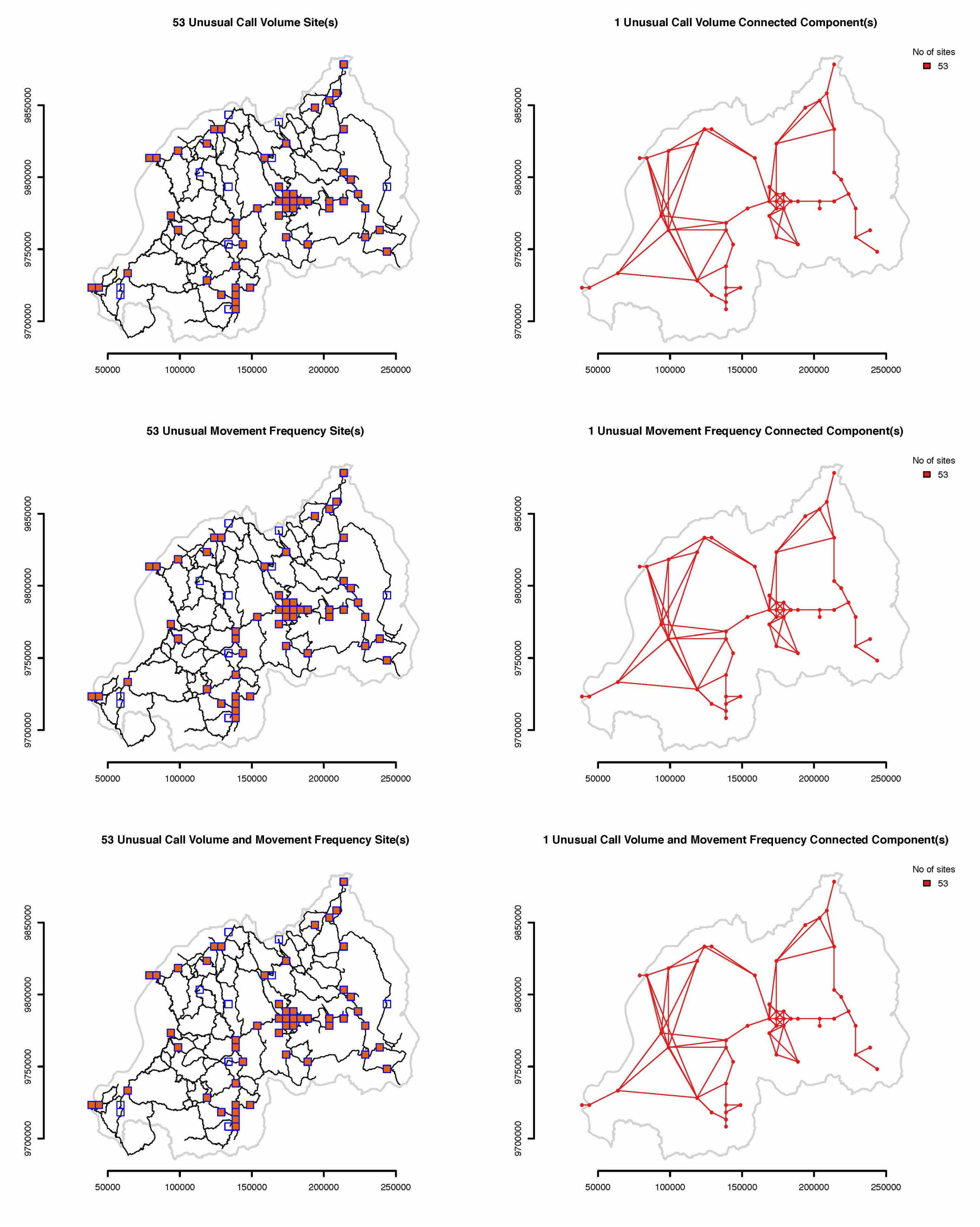

FIG. M. **Sites with unusually low behavior on September 19, 2007.** A number of 53 sites recorded unusually low call volume and movement frequency. These sites belong to the same spatial cluster.

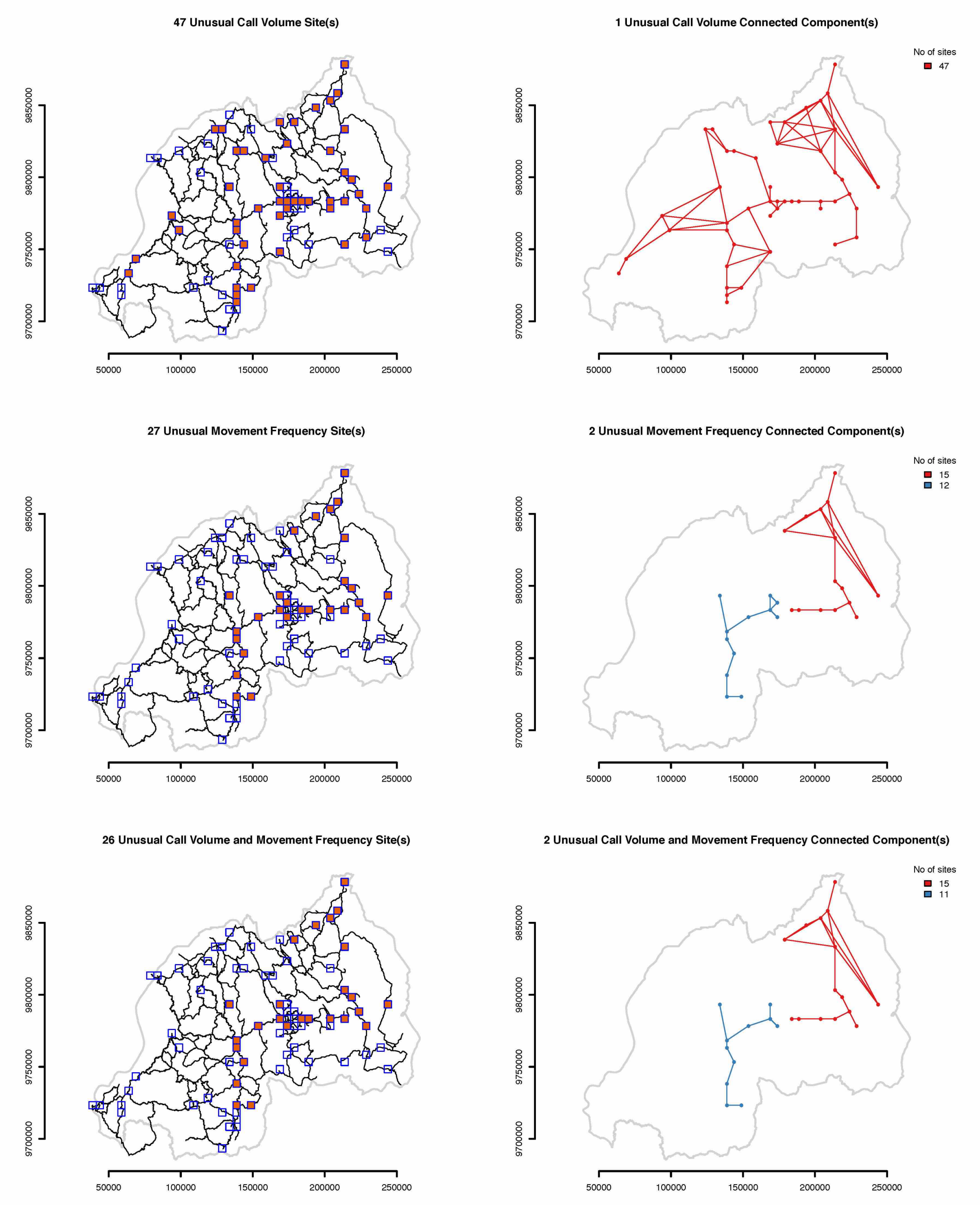

FIG. N. **Sites with unusually high behavior on December 24, 2007.** A number of 26 sites recorded unusually high call volume and movement frequency. A number of 21 additional sites recorded unusually high call volume, while one other site recorded unusually high movement frequency. The sites in these three groups belong to one or two spatial clusters.

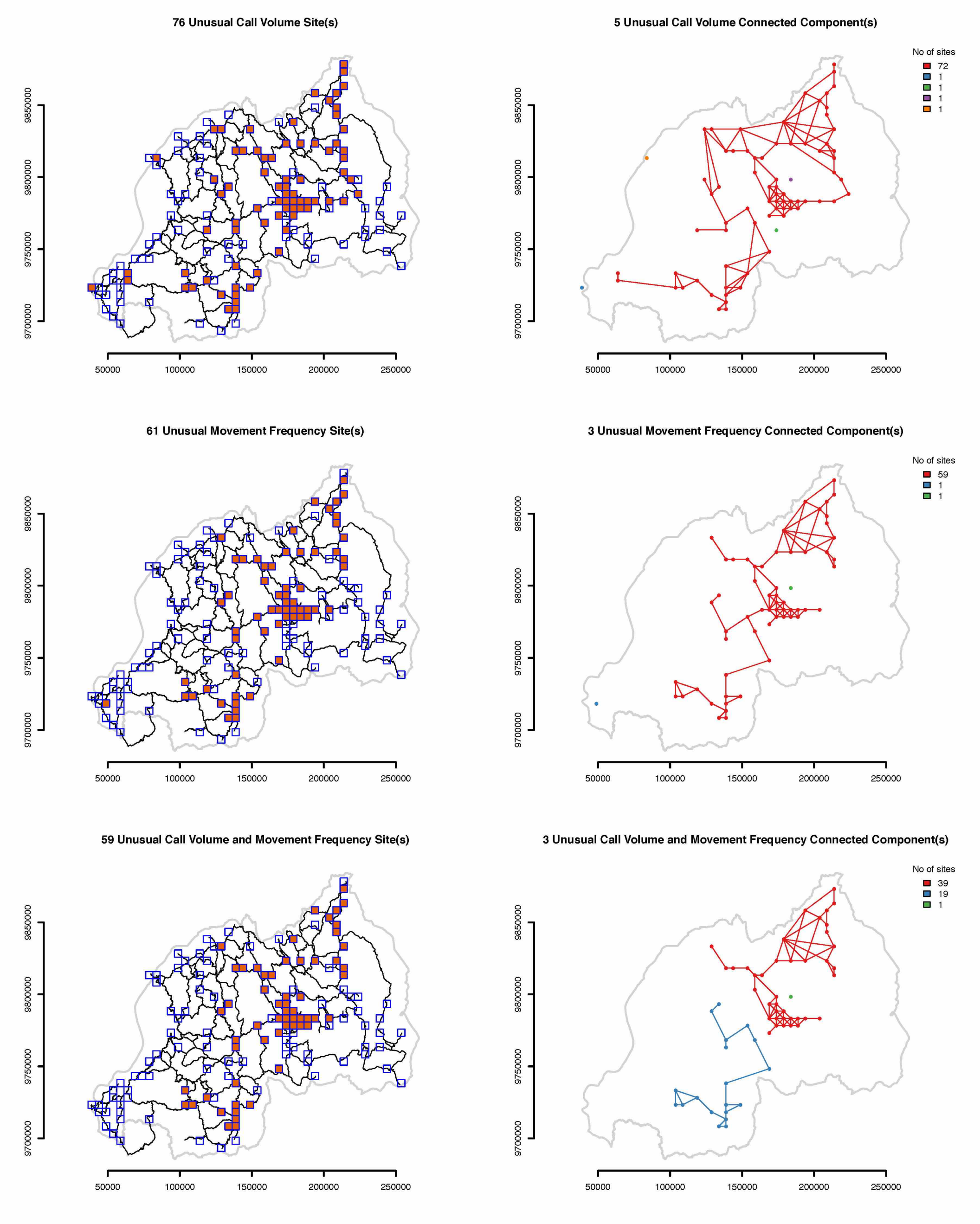

Fig. O. **Sites with unusually high behavior on December 24, 2008.** A number of 59 sites recorded unusually high call volume and movement frequency. A number of 17 additional sites recorded unusually high call volume, while two other sites recorded unusually high movement frequency. Most of the sites in these three groups belong to one or two spatial clusters, but small spatial clusters with only one site are also present.

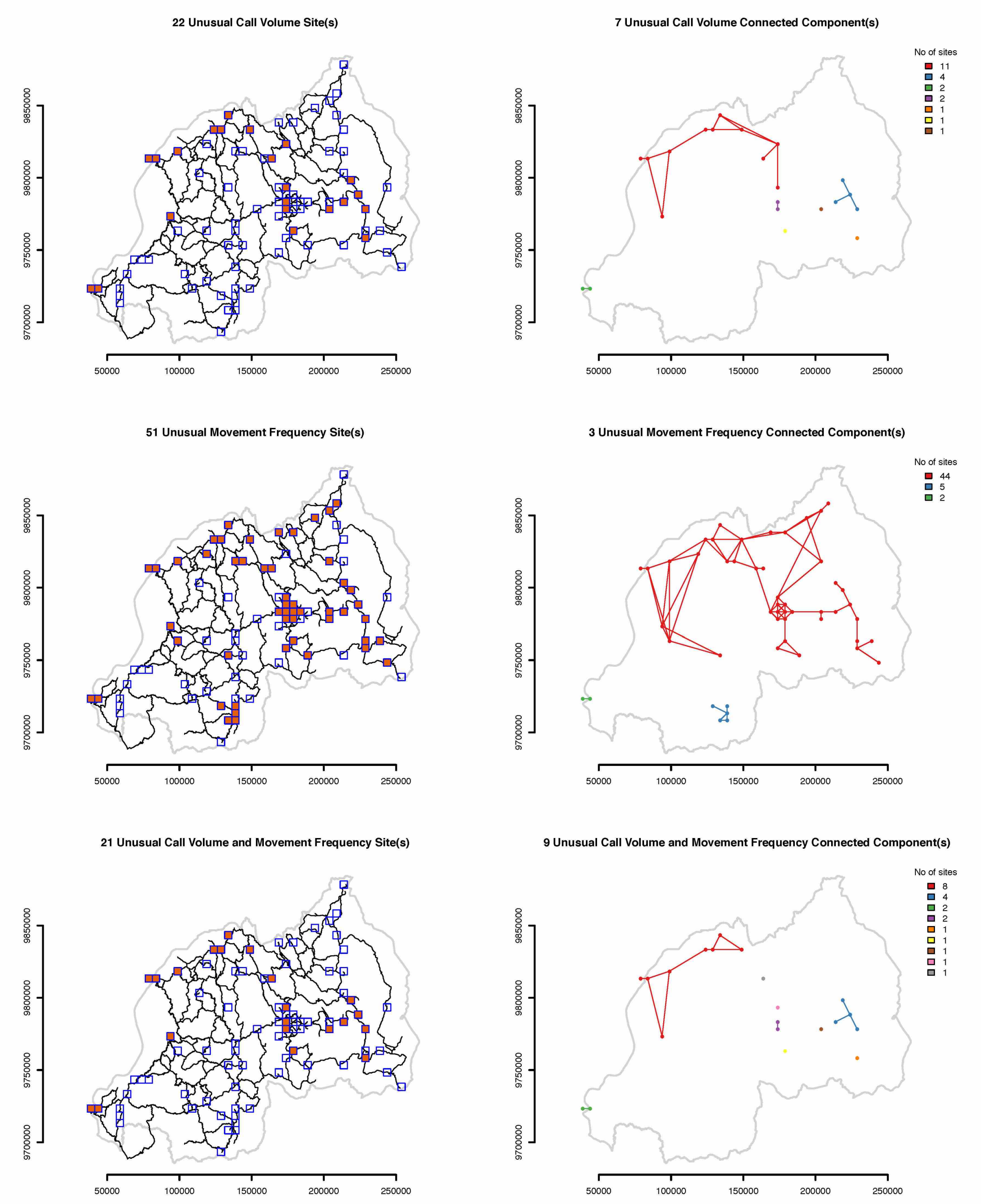

Fig. P. **Sites with unusually high behavior on January 1, 2008.** A number of 21 sites recorded unusually high call volume and movement frequency. One additional site recorded unusually high call volume, while 30 other sites recorded unusually high movement frequency. Most of the sites in these three groups belong to one spatial cluster. Smaller spatial clusters with up to four sites are also present.

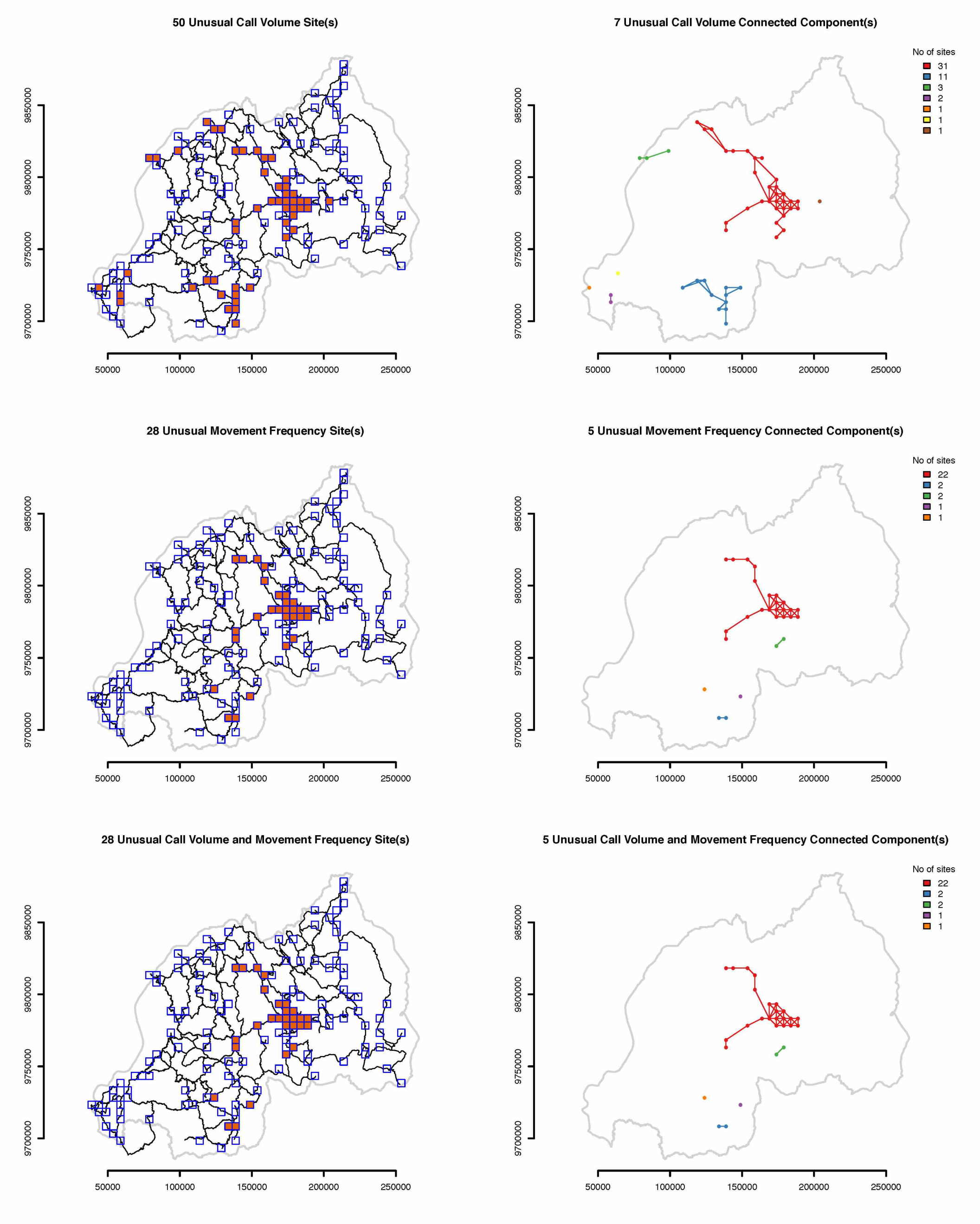

Fig. Q. **Sites with unusually high behavior on December 31, 2008.** A number of 28 sites recorded unusually high call volume and movement frequency. A number of 22 additional sites recorded unusually high call volume. Most of the sites in these three groups belong to one or two spatial clusters. Smaller spatial clusters with one or two sites are also present.

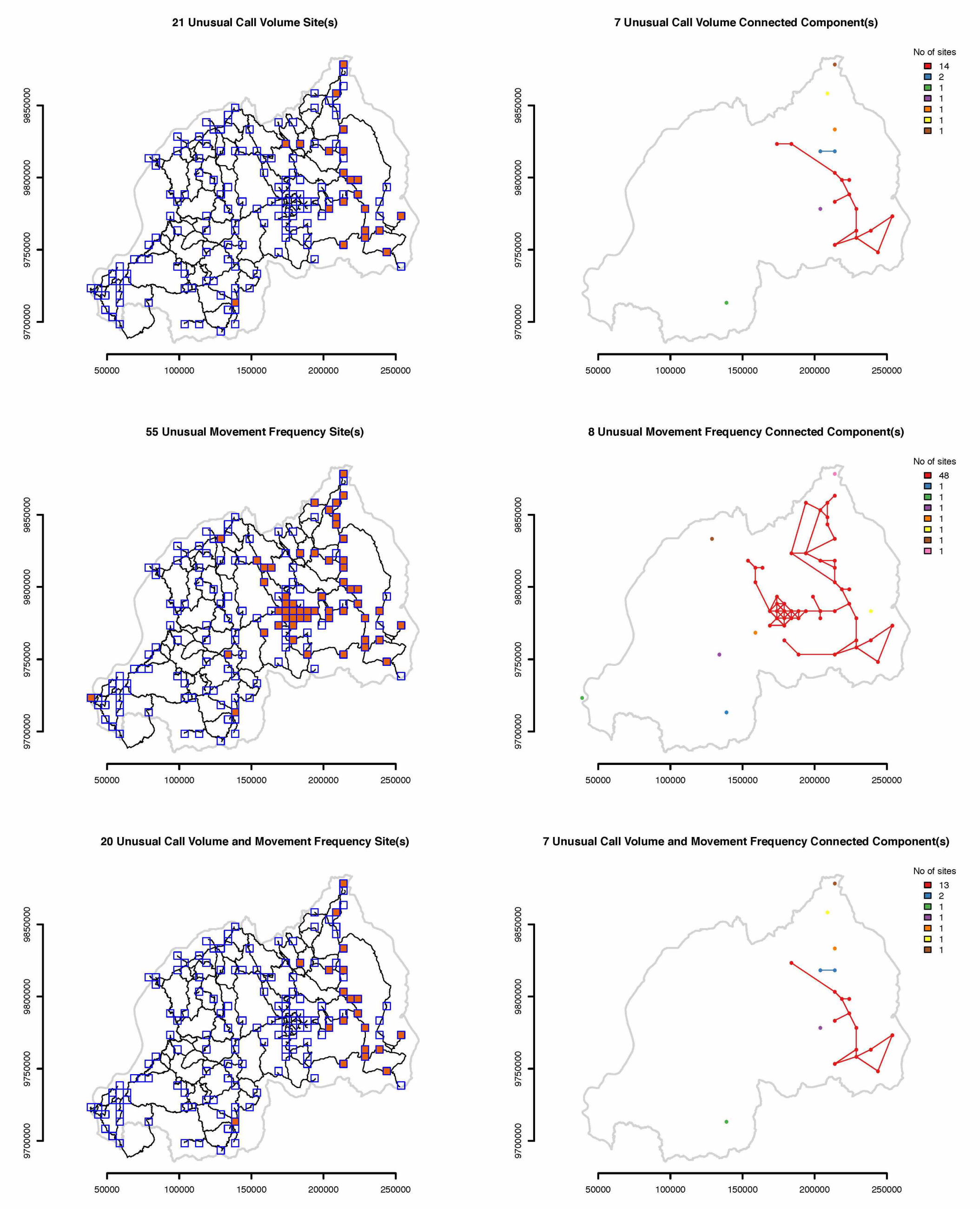

FIG. R. **Sites with unusually high behavior on January 1, 2009.** A number of 20 sites recorded unusually high call volume and movement frequency. One additional site recorded unusually high call volume, while 35 other sites recorded unusually high movement frequency. Most of the sites in these three groups belong to one spatial cluster. Smaller spatial clusters with one or two sites are also present.

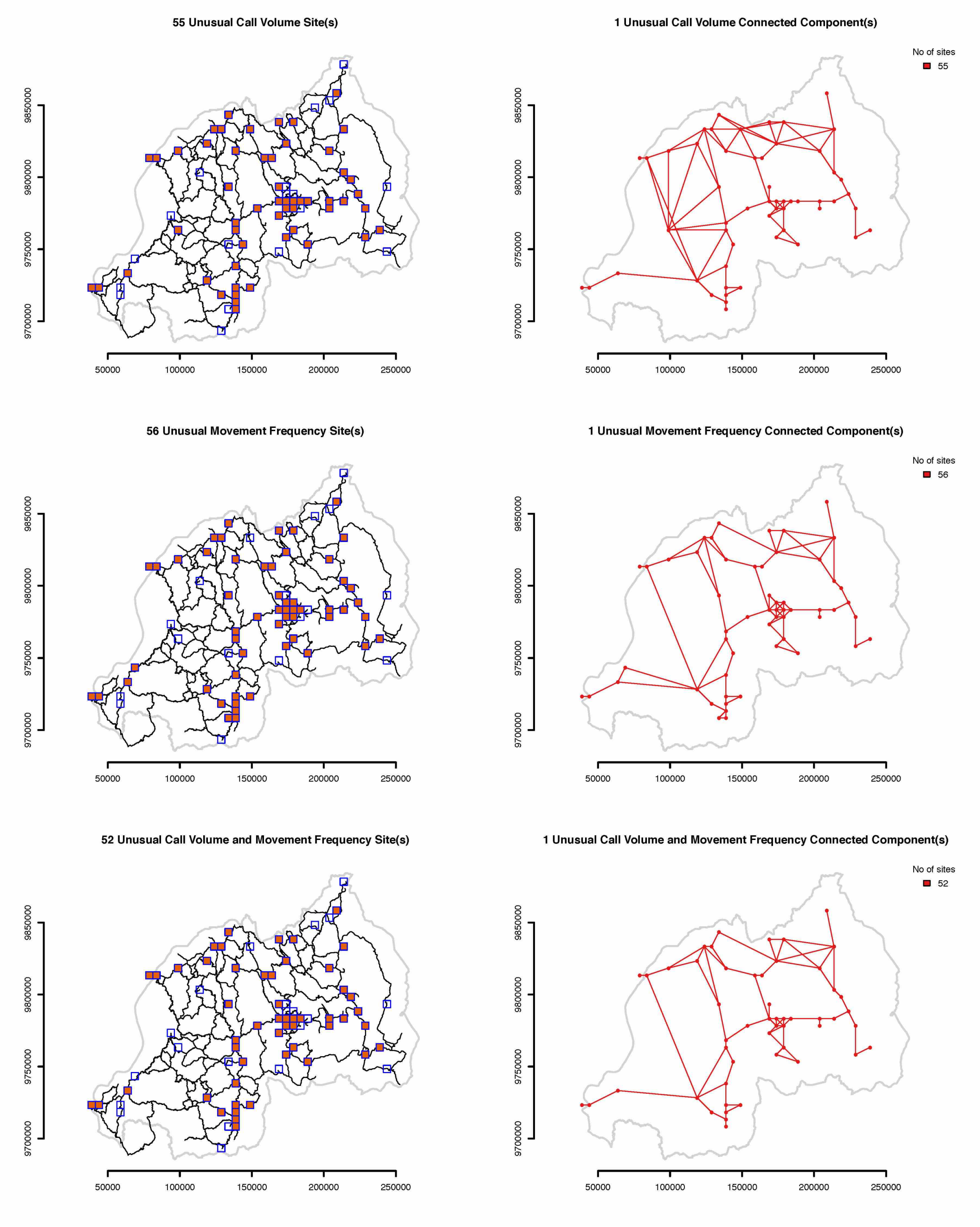

Fig. S. **Sites with unusually high behavior on November 9, 2007.** A number of 52 sites recorded unusually high call volume and movement frequency. Three additional sites recorded unusually high call volume, while one other site recorded unusually high movement frequency. The sites in these three groups belong to one spatial cluster.

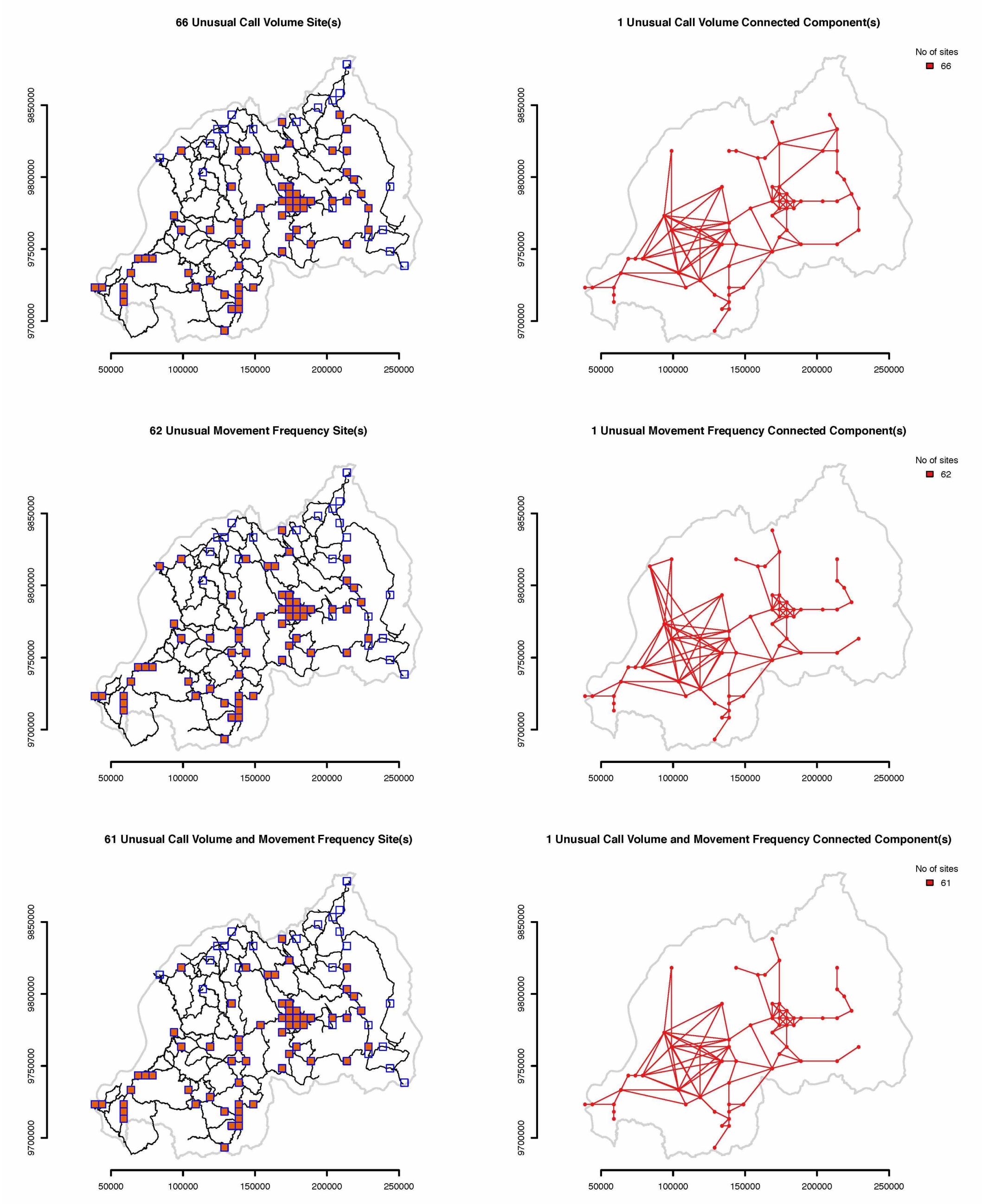

Fig. T. **Sites with unusually low behavior on April 24, 2008.** A number of 61 sites recorded unusually low call volume and movement frequency. Five additional sites recorded unusually low call volume, while one other site recorded unusually low movement frequency. The sites in these three groups belong to one spatial cluster.

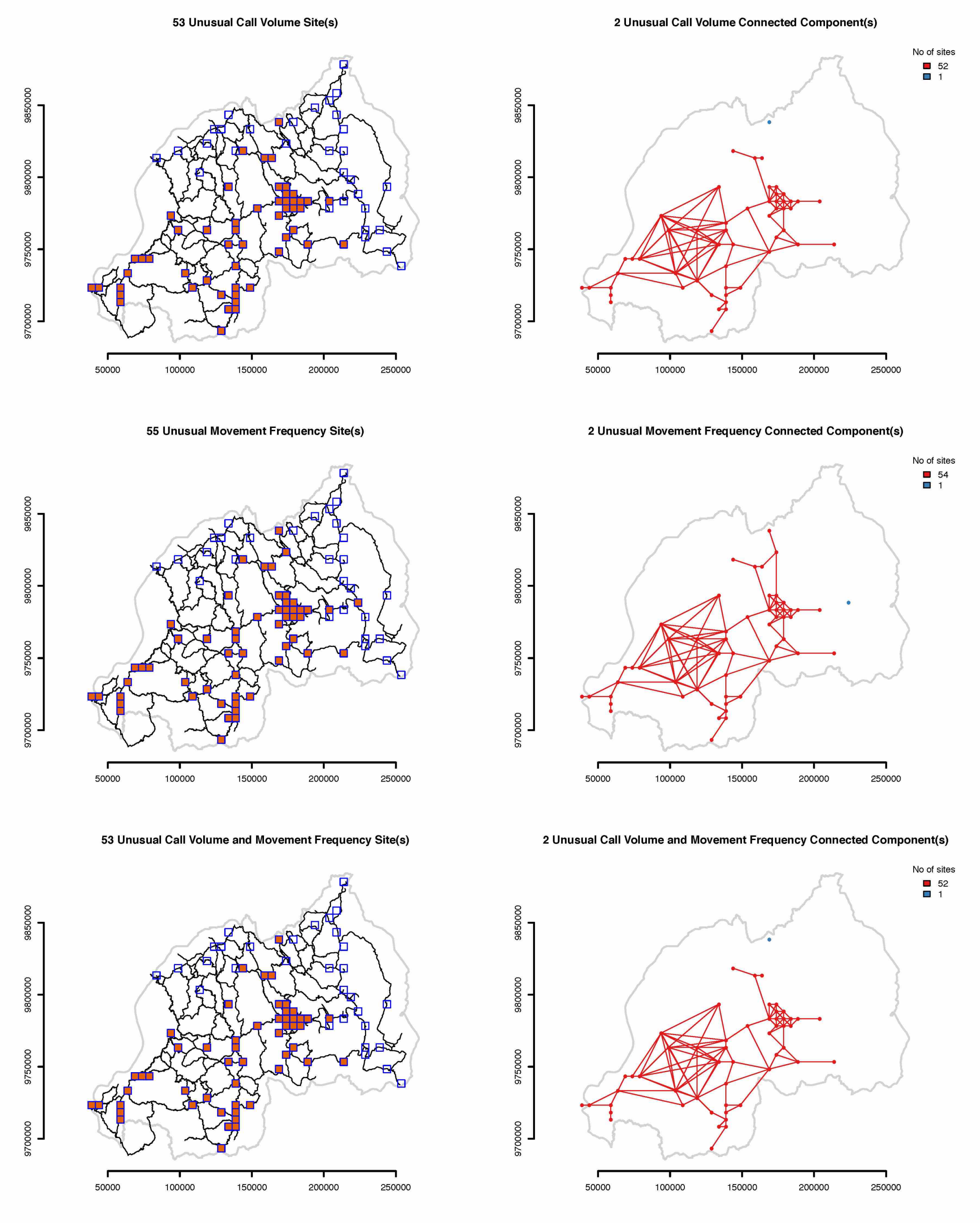

FIG. U. **Sites with unusually low behavior on April 25, 2008.** A number of 53 sites recorded unusually low call volume and movement frequency. Two additional sites recorded unusually low movement frequency. The sites in these two groups belong to one large spatial cluster and to another small spatial cluster with just one site.

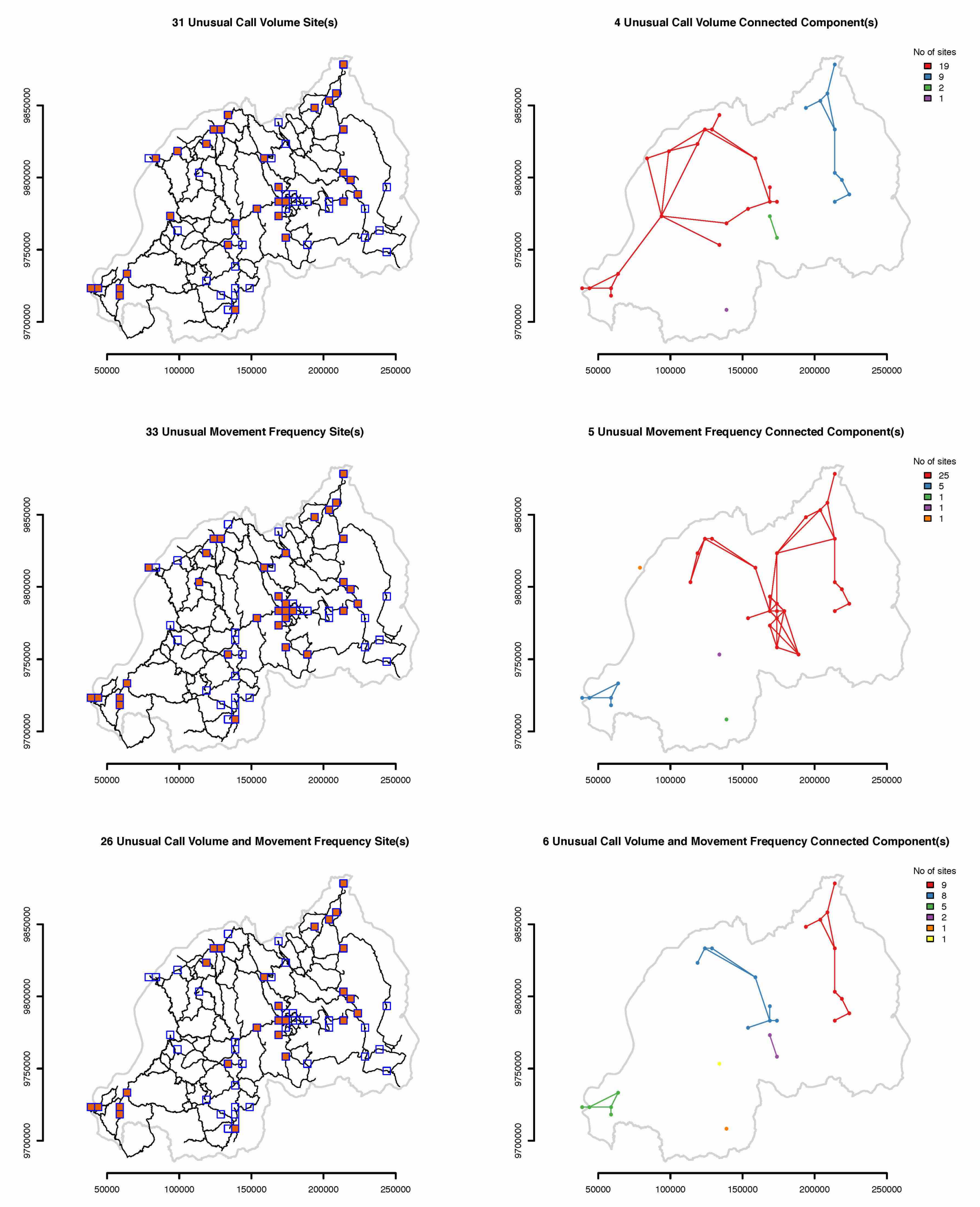

Fig. V. **Sites with unusually low behavior on April 7, 2007.** A number of 26 sites recorded unusually low call volume and movement frequency. Five additional sites recorded unusually low call volume, while 7 other sites recorded unusually low movement frequency. Most of the sites in these three groups belong to two spatial clusters. Smaller spatial clusters are also present.

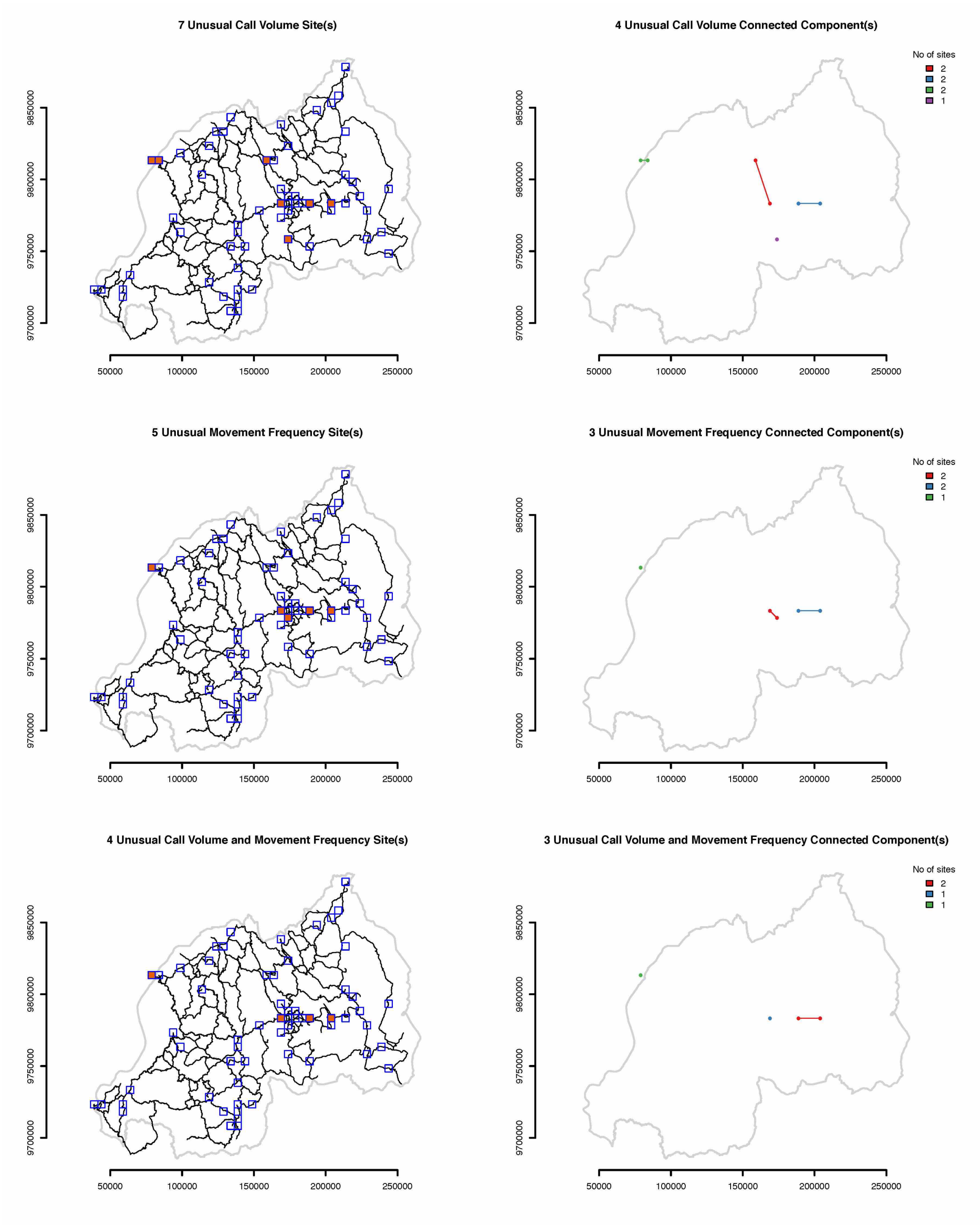

Fig. W. **Sites with unusually low behavior on April 8, 2007.** Four sites recorded unusually low call volume and movement frequency. Three additional sites recorded unusually low call volume, while one other site recorded unusually low movement frequency. The sites in these three groups belong to spatial clusters of size 1 or 2.

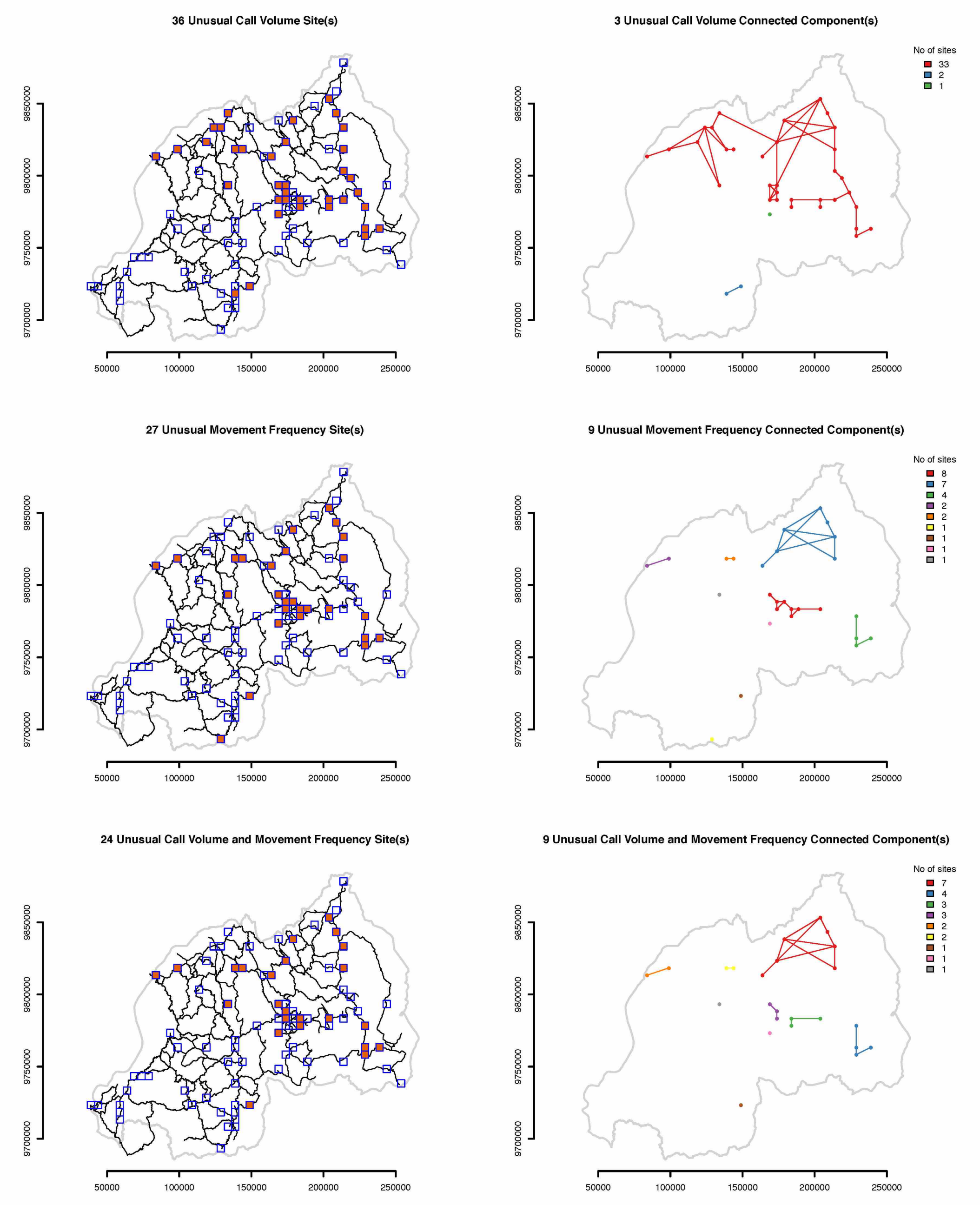

Fig. X. **Sites with unusually low behavior on April 7, 2008.** A number of 24 sites recorded unusually low call volume and movement frequency. Twelve additional sites recorded unusually low call volume, while 3 other sites recorded unusually low movement frequency. Most of the sites in these three groups belong to one spatial cluster. Smaller spatial clusters are also present.

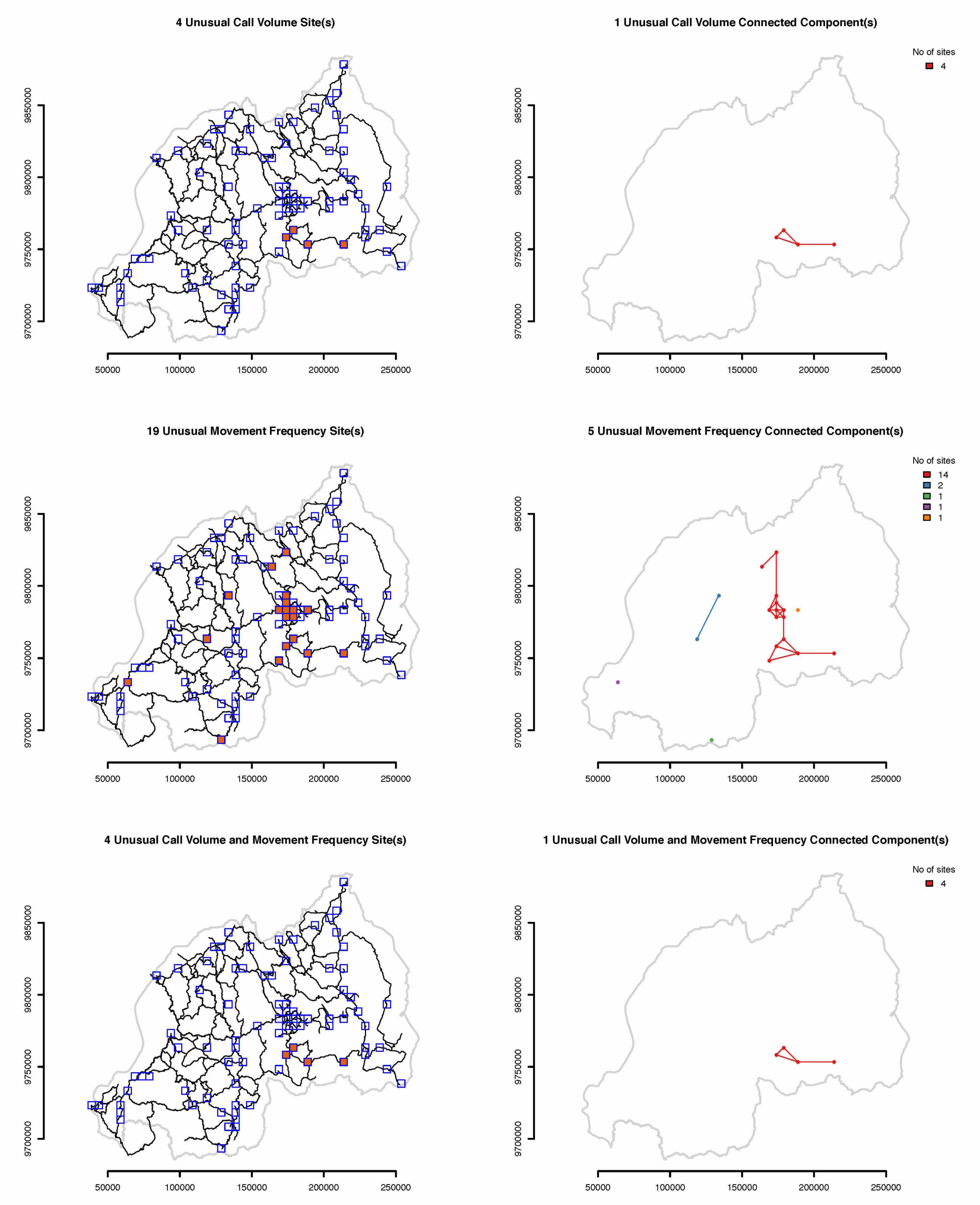

FIG. Y. **Sites with unusually low behavior on April 8, 2008.** Four sites recorded unusually low call volume and movement frequency. A number of 15 additional sites recorded unusually low movement frequency. Most of the sites in these two groups belong to one spatial cluster. Smaller spatial clusters are also present.
